# Considerations for high-yield, high-throughput cell enrichment: fluorescence versus magnetic sorting

**DOI:** 10.1038/s41598-018-36698-1

**Published:** 2019-01-18

**Authors:** Bryan A. Sutermaster, Eric M. Darling

**Affiliations:** 1 Center for Biomedical Engineering, Brown University, Providence, USA; 2 Department of Molecular Pharmacology, Physiology, and Biotechnology, Brown University, Providence, USA; 3 Department of Orthopaedics, Brown University, Providence, USA; 4 School of Engineering, Brown University, Providence, USA

## Abstract

Efficient sorting methods are required for the isolation of cellular subpopulations in basic science and translational applications. Despite this, throughputs, yields, viabilities, and processing times of common sorting methods like fluorescence-activated cell sorting (FACS) and magnetic-activated cell sorting (MACS) are underreported. In the current study, we set out to quantify the ability of these sorting methods to separate defined mixtures of alkaline phosphatase liver/bone/kidney (ALPL)-expressing and non-expressing cell types. Results showed that initial MACS runs performed using manufacturer’s recommended antibody and microbead concentrations produced inaccurate ALPL+ vs. ALPL− cell splits compared to FACS when ALPL+ cells were present in larger proportions (>~25%). Accuracy at all proportions could be achieved by using substantially higher concentrations of labeling reagents. Importantly, MACS sorts resulted in only 7–9% cell loss compared to ~70% cell loss for FACS. Additionally, MACS processing was 4–6 times faster than FACS for single, low proportion samples but took similar time for single, high-proportion samples. When processing multiple samples, MACS was always faster overall due to its ability to run samples in parallel. Average cell viability for all groups remained high (>83%), regardless of sorting method. Despite requiring substantial optimization, the ability of MACS to isolate increased cell numbers in less time than FACS may prove valuable in both basic science and translational, cell-based applications.

## Introduction

Cell sorting, enrichment, and purification methods are powerful tools enabling the isolation of cellular subpopulations for basic science and clinical applications. The stromal vascular fraction (SVF), or vascular-associated cellular component, of lipoaspirate has been identified as an attractive cell source for both basic science and translational study as it contains subpopulations of adipose-derived stem cells (ASCs) and other progenitors^1,2^. Compared to other stem cell niches like bone marrow and muscle, adipose tissue contains higher percentages of differentiable cells, and can be isolated with ease and little donor site morbidity^3^. As the SVF is comprised of a heterogeneous cell population, *in vitro* plating/expansion or cell separation techniques are required to isolate ASCs from non-stem cell types^4–6^. Plating and expansion is a time consuming process not compatible with single-surgery procedures. More rapid cell separation techniques are needed for time-sensitive applications.

Subpopulations of ASCs and other progenitors can be fluorescently tagged based on biochemical markers and subsequently isolated from other cell types in the SVF by cell sorting techniques^7–12^. The gold standard for cell separation is fluorescence-activated cell sorting (FACS). While FACS is capable of processing millions of cells and isolating multiple, high purity subpopulations, it is also relatively time consuming for very large cell numbers and requires expensive machinery. A related technology, magnetic-activated cell sorting (MACS), relies on direct (primary antibody-conjugated microbead) or indirect (primary antibody plus secondary antibody-conjugated microbead) magnetic labeling of cells prior to separation in a magnetic field^13^. MACS is also used to select for cell populations using surface markers but is less time consuming and requires less expensive equipment than FACS. However, it lacks the sensitivity and cell-specific data provided by a fluorescence-based system and is not easily compatible with multiple-marker profiles. Surprisingly, measures of cell throughput and yield, viabilities, and processing time between FACS and MACS are largely unreported, making it difficult to compare the practicality of the two techniques for a given application.

Cell separation techniques for ASCs often employ multiple surface markers to specifically define the cell type, as a single, definitive marker has yet to be identified^14,15^. A general ASC definition proposed by the International Federation of Adipose Therapeutics and Science (IFATS) includes positive/negative expression for four surface markers (CD34+/CD31−/CD45−/CD235a−), with an additional four markers for increased specificity (CD13, CD73, CD90, and CD105)^15^. These restrictive definitions result in very small numbers of enriched, yet still heterogeneous, cells such that the population input to FACS must be extremely large to acquire therapeutically relevant numbers (~10^6^–10^8^) as output^16–22^. Less restrictive surface marker profiles may enable isolation of larger cell populations and prove advantageous for regenerative medicine applications. One such marker, alkaline phosphatase liver/bone/kidney (ALPL), is a membrane bound protein involved in early matrix mineralization during osteogenesis and may be a useful target for identifying stem cell subpopulations, particularly for end applications of bone regeneration^23–28^. Previously, groups have isolated subpopulations of induced pluripotent stem cells and jaw periosteal cells based on ALPL expression that were capable of increased osteogenesis, though this has not yet been demonstrated with primary SVF cells^29,30^.

The objective of this study was to quantify the processing times, cell yields and viabilities of MACS and FACS separations using defined mixtures of osteogenically primed SVF cells and A375 human melanoma cells based on their expression of ALPL. To accomplish this, primary SVF cells were first expanded and osteogenically stimulated to upregulate expression of the ALPL marker in responsive cell types. After priming, SVF cells were mixed in defined ratios with A375 cells (0:1, 1:3, 1:1, 3:1, 1:0) and separated based on ALPL expression using FACS or MACS. Processing time and cell throughput, yield, and viability for ALPL+ and ALPL− groups were quantified and compared between the two sorting methods. Effort was made to identify and reconcile discrepancies between the two approaches to better inform researchers using these techniques for cell enrichment/purification studies.

## Methods

### SVF Isolation and Culture

Primary, human lipoaspirate was procured from the breast and abdomen of one, informed and consenting, female donor (56 yo, prior diagnosis: lipodystrophy) in accordance with guidelines approved by the Institutional Review Board of Rhode Island Hospital. Stromal vascular fraction (SVF) cells were isolated according to established protocols and immediately placed in cryogenic storage before use in this study^6^.

For all FACS and MACS sorts, freshly thawed, passage 0 (P0) SVF cells were maintained in expansion medium with medium changes every other day until >90% confluent in T182 tissue culture flasks (Genesee Scientific, San Diego, CA). Expansion medium maintained cells in a proliferative, multipotent state and consisted of Dulbecco’s modified Eagle’s medium: nutrient mixture F-12 (DMEM-F12) (GE Healthcare HyClone, Pittsburgh, PA), 10% fetal bovine serum (FBS, ZenBio, Research Triangle Park, NC), 5 ng/mL epidermal growth factor, 1 ng/mL fibroblast growth factor, 0.25 ng/mL transforming growth factor-β1 (R&D Systems, Minneapolis, MN), and 1X antibiotic/antimycotic (anti/anti, GE Healthcare HyClone)^31^. Once at near confluence, cells were primed with osteogenic medium for four days prior to sorting procedures. Osteogenic medium consisted of DMEM with high glucose (DMEM-hg, GE Healthcare HyClone), 10% FBS (ZenBio), 10 mM β-glycerophosphate, 150 µM ascorbate-2-phosphate, 10 nM dexamethasone, 9.1 nM vitamin-D3 (Sigma-Aldrich, St. Louis, MO), and 1X anti/anti (GE Healthcare HyClone). Concurrently, freshly thawed A375 human melanoma cells were maintained in growth medium, with medium changes every other day for twelve days prior to sorting procedures. A375 growth medium consisted of DMEM-F12 (GE Healthcare HyClone) with 10% FBS (ZenBio) and 1X anti/anti (GE Healthcare HyClone).

### Fluorescence-Activated Cell Sorting

Four-day, osteogenically primed, P0, ALPL+SVF cells and ALPL− A375 cells were detached from flasks using Accutase cell detachment solution (Corning Life Sciences, Tewksbury, MA) and filtered through 40 µm cell strainers (Fisher Scientific, Hampton, NH). Viability and cell numbers were determined using a trypan blue exclusion method. 8 × 10^6^ cell mixtures were made according to the following ALPL+:ALPL− cell ratios: 0:1, 1:3, 1:1, 3:1, and 1:0. Cell mixtures were pelleted, and resuspended in 110 µL of 1:11 dilutions of anti-ALPL−allophycocyanin (APC) stock antibody solution for 10 min at 4 °C, according to manufacturer’s protocol (Clone: W8B2, Catalogue Number: 130-093-589, Miltenyi Biotec GmbH, Bergisch Gladbach, Germany). After labeling, cells were maintained on ice until sorted.

To lessen the impact of user variability, a single, expert operator with over 20 years of fluorescence sorting experience performed all FACS sorts using a stream-in-air BD Influx cell sorter fitted with a 100 µm nozzle and 488 nm trigger and 633 nm fluorescence excitation lasers (Becton Dickinson, Franklin Lakes, NJ). Sorts were performed using a 20.1 psi driving pressure and a droplet frequency of 38.15 kHz. Though droplet delay was set independently for each sort, a typical value from a representative experiment was 28.9967 ms (1/droplet frequency). FSC and SSC gates and laser gains were established using two, 8 × 10^6^ cell, unlabeled (A375 and osteo-primed SVF, respectively) and two, 8 × 10^6^ cell, anti-ALPL-APC-labeled (A375 and osteo-primed SVF, respectively) samples in 500 µL autoMACS rinsing solution (Miltenyi Biotec) with 0.5% BSA (Sigma-Aldrich, St. Louis, MO) after filtering through 40 µm cell strainers. Subsequently, 8 × 10^6^ ALPL+:ALPL− cell mixtures of the ratios described above were passed through 40 µm cell strainers and sorted into ALPL+ and ALPL− fractions. 50 mL conical collection tubes (Genesee Scientific) containing 5 mL of cold, DMEM-hg base medium were used to preserve post-sort viability. After sorting, cells were maintained on ice until counted. Total cell yields were defined as the sum of live and dead cells counted at the output. Output fractions were calculated for both ALPL+ and ALPL− subpopulations by dividing the post-sort total cell count for each subpopulation by the sum of ALPL+ and ALPL− post-sort total cell counts. Viability was defined as the number of live output cells divided by the sum of live and dead output cells, multiplied by 100. Each five-condition run (0:1, 1:3, 1:1, 3:1, 1:0) was repeated three times.

### Magnetic-Activated Cell Sorting (Manufacturer’s Protocol)

For experimental sorting runs, four-day, osteogenically primed, P0, ALPL+SVF and ALPL− A375 cells were uplifted, filtered, and combined into mixtures of defined ratios as described above. Four unlabeled and anti-ALPL-APC-labeled control conditions were prepared as described previously. The remaining 8 × 10^6^ cell experimental conditions (0:1, 1:3, 1:1, 3:1, and 1:0 ALPL+:ALPL−) were antibody-labeled as previously described, pelleted, and subsequently labeled with 100 µL of 1:5 dilution, anti-APC microbeads (Catalogue Number: 130-090-855, Miltenyi Biotec) for 15 minutes at 4 °C. Each 8 × 10^6^ antibody- and microbead-labeled condition was magnetically sorted at room temperature using “MS” columns, inserted into a Miltenyi OctoMACS separator (Catalogue Numbers: 130-042-201 and 130-042-109, respectively, Miltenyi Biotec). Post-sort retained and flow-through fractions from each experimental condition were counted, and viabilities determined as described previously. After sorting, cells were maintained on ice until counted. Output fractions, retained and flow-through yield, and viability were calculated as described above. After counting, all retained and flow-through fractions for each experimental condition were further analyzed by flow cytometry to quantify the distribution of ALPL+ and ALPL− cells within each fraction/condition. FSC and SSC gates and laser gains were established using the four, labeled and unlabeled control conditions, as described previously. Each five-condition run (0:1, 1:3, 1:1, 3:1, 1:0) was repeated three times.

### Magnetic-Activated Cell Sorting Optimization

Optimization experiments were performed to reconcile discrepancies between MACS-isolated retained and flow-through fractions and the defined ALPL+ and ALPL− cell splits. Antibody and microbead labeling concentrations were varied to determine their effect on retained and flow-through output fractions and percent cell yield using only fixed, ALPL+SVF cells. Primary SVF cells were expanded and osteogenically primed as described previously. After four days of priming, cells were detached from flasks using Accutase, pelleted, and fixed in suspension for 15 min in 10% formalin phosphate (Fisher Scientific) at room temperature. To determine the influence of microbead concentration, five aliquots of 8 × 10^6^ fixed, ALPL+SVF cells were labeled with 1:11 antibody dilutions, followed by 1:16, 1:8, 1:5, 1:2, or pure anti-APC microbead solutions prior to magnetic sorting using MS columns fitted with 70 µm filters (Miltenyi Biotec). Similarly, to determine the influence of antibody concentration, four aliquots of 8 × 10^6^ fixed, ALPL+SVF cells were labeled with 1:11, 1:7.5, 1:5, or 1:2.5 anti-ALP-APC antibody dilutions prior to labeling with 1:5 microbead dilutions and MACS processing using MS columns fitted with 70 µm filters. Finally, to examine the effect of simultaneously varying antibody and microbead concentration, two 8 × 10^6^ ALPL+SVF aliquots were labeled with 1:2.5 antibody dilutions followed by a 1:5 dilution or pure microbead solution and processed by MACS using MS columns fitted with 70 µm filters. Following all magnetic sorts, cells were counted and calculations made for retained and flow-through output fractions and percent cell yield. Post-sort retained and flow-through fractions of antibody-varied and simultaneous antibody- and microbead-varied trials were further analyzed by flow cytometry to determine distributions of ALPL+ and ALPL− cells within each sample.

### Magnetic-Activated Cell Sorting (Optimized Protocol)

Three separate, five-condition (0:1, 1:3, 1:1, 3:1, 1:0 ALPL+:ALPL−) runs were performed as described previously but with increased antibody and microbead labeling concentrations. For optimized MACS protocol runs, cells were labeled with 110 µL of a 1:2.5 dilution of anti-ALPL-APC antibodies for 10 min at 4 °C, followed by 100 µL of undiluted anti-APC microbeads for 15 min at 4 °C. All post-sort counting, viability, and flow cytometric assessment was performed as described previously.

### Statistical Analysis

For MACS optimization, correlations between retained output fractions and either antibody or microbead dilution were determined by calculating Pearson’s r values on log-transformed data. For FACS and manufacturer’s and optimized protocol MACS, comparisons of total, live, and dead cell yields were made using a Kruskal-Wallis one-way analysis of variance (ANOVA) on ranks (factor: sorting method, p < 0.05). Comparisons of average viability for all ratio conditions were made using a Kruskal-Wallis one-way ANOVA on ranks (factor: sorting method, p < 0.05) to determine statistical differences between FACS (n = 25), manufacturer’s protocol MACS (n = 27), and optimized MACS (n = 27).

## Results

### MACS Optimization

**Figure 1:**
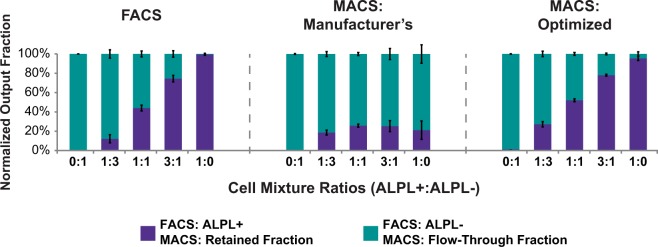
Normalized output fraction of osteogenically primed SVF and A375 controlled-ratio sorts using FACS, manufacturer’s protocol MACS, and optimized protocol MACS. Defined mixtures of osteogenically primed SVF and A375 cells were labeled with anti-ALPL-APC antibodies (and anti-APC microbeads for MACS) prior to cell sorting. After separation, ALPL+/retained and ALPL−/flow-through fractions were counted and normalized output fractions computed. For ALPL+:ALPL− cell ratios >1:3, manufacturer’s protocol MACS sorts were not able to accurately separate ALPL+ and ALPL− cells. Error bars indicate standard deviation.

**Figure 2:**
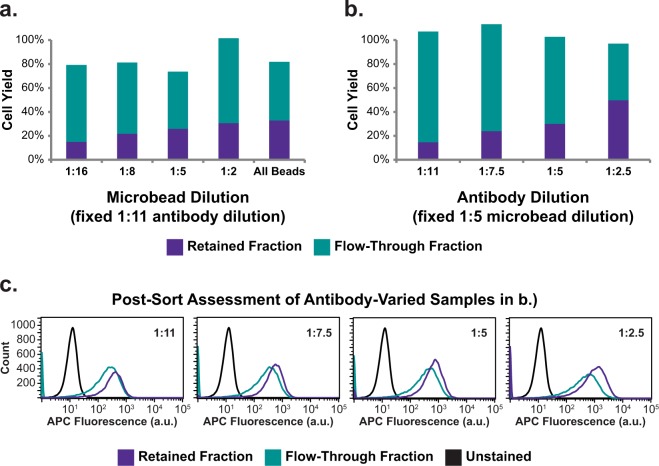
Cell yield and fraction analyses for MACS optimization trials of varying antibody and microbead concentrations. MACS cell yields of retained and flow-through fractions for pure ALPL+ optimization sorts when (**a**) microbead dilution was varied with a fixed (1:11) antibody dilution and (**b**) antibody dilution was varied with a fixed (1:5) microbead dilution. (**c**) Distributions of ALPL+ cells within retained and flow-through fractions of antibody-varied trials displayed in (**b**) were assessed via flow cytometry and confirmed a high false-negative rate in flow-through fractions. (APC fluorescence corresponds to APC dye on anti-ALPL antibody).

**Figure 3:**
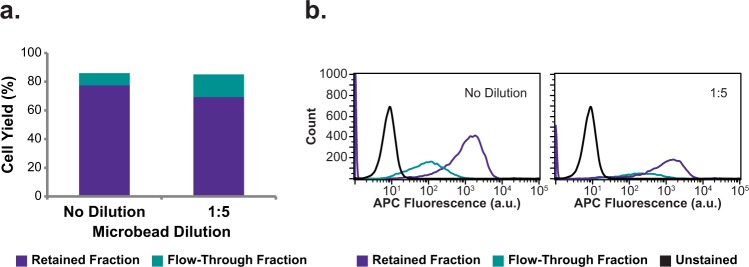
Cell yield and flow cytometric analysis for high concentration antibody and microbead MACS optimization trials. Fixed SVF cells were labeled with 1:2.5 antibody dilutions prior to 1:5 or no dilution microbead labeling and then magnetically sorted. Retained and flow-through fractions were counted and percent cell yields calculated prior to flow cytometric analysis. (**a**) Percent cell yields, (**b**) flow cytometry fluorescence histograms of post-sort fractions (APC fluorescence corresponds to APC dye on anti-ALPL antibody).

Manufacturer’s protocol MACS runs displayed major differences in retained and flow-through output fractions compared to FACS-separated ALPL+ and ALPL− fractions (Fig. 1). To determine whether antibody and microbead concentrations specified by the manufacturer’s MACS protocol was responsible for the observed differences, MACS antibody- and microbead- treatment concentrations were varied in a series of optimization trials to determine their effect on isolation of fixed, ALPL+SVF cells. Data showed that when antibody labeling concentration was held constant (1:11) and microbead concentration varied (1:16, 1:8, 1:5, 1:2, and no dilution), a very strong positive correlation existed between microbead concentration and retained cell yields for MACS optimization tests (Fig. 2a, *r* = 0.95). Similarly, in MACS optimization runs performed with varied antibody concentrations (1:11, 1:7.5, 1:5, and 1:2.5) and fixed microbead concentration (1:5), a very strong positive correlation existed between antibody concentration and retained fraction yields (Fig. 2b, *r* = 0.99). Regardless of antibody concentration, flow cytometric analysis showed that the retained and flow-through fractions all exhibited high fluorescence and large overlaps in their fluorescence histograms (Fig. 2c). This overlap suggested manufacturer’s protocol MACS-isolated flow-through subpopulations were comprised largely of false negatives. To address this complication, MACS optimization runs were performed with high antibody concentration (1:2.5) coupled with high microbead concentration (1:5 or no dilution). When performing MACS with these increased antibody and microbead concentrations, false negative rates were minimized as evidenced by low flow-through cell percentages and little fluorescence overlap between retained and flow-through fractions (Fig. 3).

**Figure 4:**
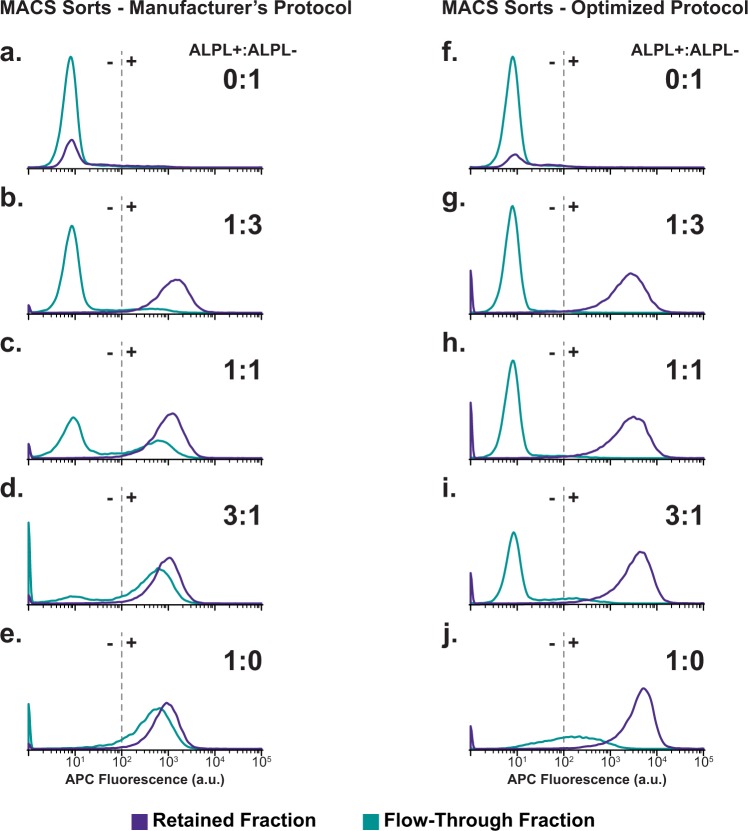
Post-MACS flow cytometric analysis of retained and flow-through fractions isolated using either manufacturer’s or optimized protocols. After MACS separation of defined mixtures of ALPL+ and ALPL− cells (0:1, 1:3, 1:1, 3:1, 1:0) according to manufacturer’s (**a**–**e**) or optimized protocols (**f**–**j**), post-sort subpopulations were assessed by flow cytometry. For input populations larger than 1:3, manufacturer’s protocol sorts exhibited large false negative rates.

A complete set of MACS runs was performed using the optimized antibody (1:2.5 dilution) and microbead (no dilution) concentrations. Retained and flow-through fractions collected with the optimized protocol mirrored both the ALPL+/ALPL− fractions observed with FACS, and the defined input fractions (Fig. 1). Flow cytometry of the retained and flow-through fractions indicated low fluorescence overlap, and thus accurate ALPL+/ALPL− subpopulation separation, compared to results using the manufacturer’s MACS protocol (Fig. 4).

### Cell Yield, Viability, and Sorting Time for Sorting Experiments

**Figure 5:**
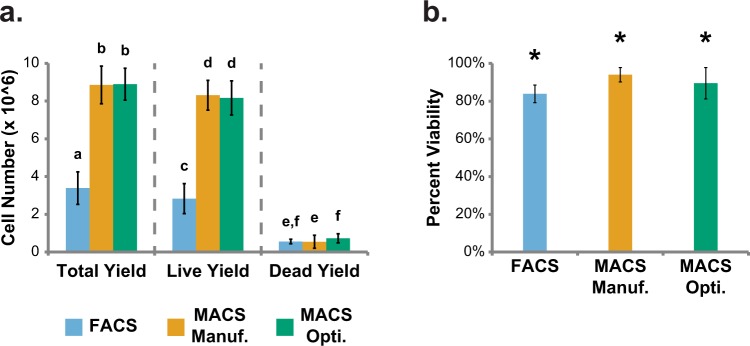
Total, live, and dead cell yields and post-sort viabilities for FACS, manufacturer’s protocol MACS, and optimized protocol MACS sorts. Post-sort (**a**) total, live, and dead cell yields and (**b**) viabilities for FACS and manufacturer’s and optimized protocol MACS (a. Unlike letters within total, live, and dead yield sections indicate statistical difference, p < 0.05; b. *Indicates significant difference in viability among sorting methods, p < 0.05). Error bars indicate standard deviation. Average live cell input cell number: 8 × 10^6^ for all sorting methods. Average total cell input number: 10.7 ± 1.4 × 10^6^ for FACS, 9.5 ± 0.8 × 10^6^ for manufacturer’s protocol MACS, and 9.8 ± 0.3 × 10^6^ for optimized protocol MACS.

The effectiveness of a cell sorting procedure depends substantially on the number of viable cells it can process. Comparisons were made between FACS and manufacturer’s and optimized protocol MACS approaches, with special focus on cell yields (total, live, and dead) and viability. On average, FACS produced low total and live cell yields (Fig. 5a). Comparatively, manufacturer’s and optimized protocol MACS produced high cell yields, exhibiting only 10% and 14% of total FACS cell loss (Fig. 5a). Dead cell yields were universally low (Fig. 5a). Likewise, average cell viability was universally high among sorting methods, though both manufacturer’s and optimized protocol MACS exhibited slightly higher viability than FACS (p < 0.0001 and p < 0.002, respectively, Fig. 5b). As a practical parameter, individual FACS procedures took 20–30 minutes, while individual manufacturer’s protocol MACS procedures took approximately 5 minutes. Individual optimized protocol MACS sorting times scaled with increasing ALPL+:ALPL− input ratios, such that small ratios (0:1 and 1:3) took ~5 minutes while large ratios (3:1 and 1:0) took ~30 minutes to complete.

## Discussion

The overarching goal of this study was to enable researchers to make informed decisions about cell sorting methods by quantifying important, vastly underreported sorting parameters including cell throughputs, yields, and viabilities of FACS and MACS procedures. To do this we created a series of defined mixtures of osteogenically primed SVF (ALPL+) and A375 human melanoma (ALPL−) cells and separated them using FACS and MACS. We hypothesized that MACS would enable faster isolation of larger numbers of highly viable cells compared to FACS. Indeed, we found that manufacturer’s protocol MACS sorts were considerably faster and lost fewer cells than FACS but were unable to accurately separate ALPL+ from ALPL− subpopulations. Optimization necessitated substantially increased MACS antibody and microbead labeling concentrations, thereby enabling accurate separation of ALPL+ and ALPL− subpopulations, albeit at a higher cost-per-sort. Optimized-protocol MACS procedures were completed 0–6 times faster than FACS per sort, with larger ALPL+:ALPL− mixtures taking progressively longer than smaller ratio mixtures.

While FACS runs confirmed our preconceptions of fluorescence-based sorting (i.e., high purity, low throughput and yield), comparative manufacturer’s protocol MACS runs yielded contradictory results as flow-through fractions exhibited high false negative rates.. Though manufacturer’s protocol MACS provided a higher percent total cell yield (93% ± 8% versus 32% ± 11%, p < 0.0001) and percent live cell yield (104% ± 10% versus 35% ± 10%, p < 0.0001), its retained- and flow-through-normalized output fractions diverged from the defined input values for the higher, osteo-primed SVF:A375 ratios (1:1, 3:1, and 1:0, Fig. 1). The larger than anticipated flow-through normalized output fractions suggested that manufacturer’s protocol MACS-isolated flow-through groups were primarily composed of false negative cells. These suspicions were confirmed upon flow cytometric analysis of the retained (nominally “ALPL+”) and flow-through (nominally “ALPL−”) fractions. The fluorescence histograms for these fractions exhibited overlapping, high fluorescence values, indicating inadequate separation of highly fluorescent, ALPL+ and non-fluorescent, ALPL− cells. These results are consistent with a previous study, which demonstrated high (16–35%) false-negative rates with manufacturer’s protocol MACS separations^32^. Our subsequent hypothesis was that the manufacturer-recommended MACS protocol provided insufficient antibodies and/or microbeads to sufficiently label a highly expressed surface protein like ALPL across large numbers of cells. Insufficient amounts of antibody could result in too few surface ALPL proteins being antibody-labeled, thereby limiting the potential binding partners for magnetic microbeads that are required to hold cells in the MACS column. Similarly, insufficient numbers of microbeads could result in incomplete saturation of available antibody-labeled ALPL surface proteins, preventing cells from being retained in the column.

To determine whether the manufacturer’s protocol MACS antibody and microbead treatment concentrations were responsible for inaccurate separation of ALPL+ and ALPL− cells,, antibody and microbead concentrations were varied systematically prior to sorting in a series of optimization experiments on pure, fixed, ALPL + SVF cells. Solely increasing antibody or microbead labeling concentration was insufficient to isolate the pure, ALPL+ input cell population. However, by coupling increased antibody concentration (1:2.5) with increased microbead concentration (1:5 or no dilution), percent-retained cell yield could be brought within 10% of the defined, ~100% input population. As such, we concluded that the manufacturer’s protocol antibody and microbead concentrations were indeed insufficient to adequately label, and isolate high-proportion ALPL+ populations. We subsequently hypothesized these optimized MACS antibody and microbead labeling concentrations would enable accurate separation of ALPL+SVF and ALPL− A375 cells, in contrast to the manufacturer’s protocol.

Optimized protocol MACS sorts were performed on live cells using the higher antibody (1:2.5) and microbead (no dilution) concentrations, yielding retained and flow-through splits that more closely matched the defined input. Post-sort flow cytometric analysis indicated appropriate separation of ALPL+ and ALPL− fractions, as the fluorescence histograms of highly fluorescent retained (ALPL+) and lowly fluorescent flow-through (ALPL−) fractions displayed minimal overlap. Compared to FACS, optimized protocol MACS experiments displayed higher percent total cell yield (91% ± 8% versus 32% ± 11%, p < 0.0001) and live cell yield (102% ± 11% versus 35% ± 10%, p < 0.0001). The discrepancies in cell loss between manufacturer’s and optimized protocol MACS and FACS is likely due to the FSC and SSC (size and granularity) gating required by FACS. To ensure purity of the isolated subpopulations, FACS imposes strict size and granularity constraints on the assessed populations. As a result, multi-cell aggregates and debris are rejected during sorting, lowering yield but maintaining the purity of the output streams. MACS does not impose size and granularity constraints, thereby increasing yield at the potential expense of purity. Discrepancies in cell yield were not the only differences exhibited by FACS and MACS, however.

FACS sorts took considerably longer (~20–30 min per sort) to complete than MACS manufacturer’s protocol sorts (~5 min per sort). Processing time for MACS optimized protocol sorts varied with the proportion of ALPL+ cells in the sample; quick for lower proportions and slow for higher proportions (~5–30 minutes per sort). This characteristically slower processing time for FACS is due to the single-cell assessment approach inherent in the technology. While FACS systems can use higher fluid (air or liquid) flow rates to increase the speed of cell sorting, it is still a relatively slow process compared to MACS, which is accomplished as a bulk separation. While relatively modest cell numbers were sorted in this study (8 × 10^6^ cells), accurately separating larger, clinically relevant cell numbers with FACS would take progressively longer. Additionally, FACS sorts must be performed serially, while MACS sorts can be performed in parallel. As a result, the total time required for five, serial FACS sorts (~1.7–2.5 hrs) vastly exceeded the time required for the five, parallel MACS sorts (~5 min for manufacturer’s protocol, ~30 min for optimized protocol). Furthermore, the ready parallelization afforded by MACS allows for vast increases in input cell numbers without appreciable increases in sorting time.

Longer sorting times were required for optimized protocol MACS sorts with larger ALPL+:ALPL− sorting ratios. As cells proceed through the MACS column, magnetically labeled cells are sequestered, and the void volume of the column is effectively reduced, requiring unlabeled cells and fluid to take a slower, more circuitous path to the outlet. Thus, as ALPL+:ALPL− ratios increased so did the sorting times. This knowledge may be useful, especially when separating cells without prior knowledge of the number of positive cells in the population. By measuring the approximate sorting time, a researcher can get an estimate of the percentage of positive cells in the population. Additionally, sorting times for populations containing high percentages of positive cells could likely be reduced by diluting the population and simultaneously sorting through multiple MACS columns.

In addition to the anticipated differences in throughputs, yields, and sorting times, manufacturer’s and optimized protocol MACS displayed slightly higher, post-sort viability compared to FACS (94% ± 4% and 90% ± 8% versus 84% ± 5%, p < 0.0001 and p = 0.002, respectively), consistent with published reports^32,33^. In FACS, the high fluid flow rates coupled with small nozzle diameters have the potential to reduce cellular viability due to high shear stresses^34^. Conversely, MACS is a gravity-driven process that does not impart high shear stresses, which should help maintain good viability. In both sorting methods, keeping the cells on ice both pre- and post-sort better maintained viability as it effectively lowered the metabolic rate of the cells.

The current study used single, representative FACS and MACS systems to investigate the general performance of these technologies as measured by cell throughput, yield, and viability. That said, results will likely vary for different device operators and equipment choices (i.e., alternative cell sorter models or magnetic bead suppliers). In the current study, user-to-user variability and equipment differences were controlled for by having a single, experienced operator use equipment from a single manufacturer (BD Influx for FACS sorts, Miltenyi Biotec MACS beads and OctoMACS magnet for MACS sorts). While not conducted as part of the current study, we have completed similar FACS runs with different operators and cell sorters (BD FACS Aria and BioRad S3) for general comparison purposes. Minor variations existed, but the overall trends related to cell throughput, yield, and viability were conserved. Many different options exist for immunolabeled, magnetic cell sorting beads, like Dynabeads (Thermo Fisher) or EasySep (Stem Cell Technologies), but direct comparisons of the technologies are complicated by the proprietary nature of their characteristics (e.g., particle size, antibody density, magnetic moment, etc.). However, each of these technologies is likely to have similar limitations to Miltenyi’s MACS system, necessitating a rigorous optimization as presented in the current study to ensure sorting accuracy regardless of magnetic bead supplier. As such, though the specific throughputs, yields, and sorting times of different FACS and MACS equipment will likely vary, the relative comparison between fluorescence- and magnetic-based sorting observed in the current study is expected to remain consistent.

In conclusion, we demonstrated that MACS performed according to optimized protocols enabled a more rapid, higher-yield, and higher-viability isolation of osteo-primed, ALPL+SVF cells in our stem cell isolation model compared to FACS. As such, MACS may be the more attractive option when selecting for low-abundance cell populations or time-sensitive clinical samples intended for cell therapy applications. In fact, this has been demonstrated by clinical trials using magnetic bead-based, FDA-approved CliniMACS and ISOLEX 300i CD34 + cell isolation systems for treatment of leukemia and other hematologic diseases^35–37^. Moving forward, it will be important to study the impact that parallelized, high-throughput sorting may have in a broader range of clinical applications. Increasing the efficiency of cell separation will enable isolation of larger cell numbers in less time than current methods, an asset for both basic science and cell-based therapies.

### Major Takeaways

- MACS processes cells more quickly and with greater yields than FACS.
- Selecting for highly abundant subpopulations with MACS requires substantially more antibodies and magnetic microbeads than recommended by manufacturer protocols.
- The occurrence of false negatives in MACS, a commonly noted limitation, can be lessened by antibody/microbead optimization.
